# From worms to fish to mice

**DOI:** 10.7554/eLife.57481

**Published:** 2020-05-06

**Authors:** Guy M Benian, Hyojung J Choo

**Affiliations:** 1 Department of Pathology, Emory UniversityAtlantaUnited States; 2 Department of Cell Biology, Emory UniversityAtlantaUnited States

**Keywords:** RYR1, myopathy, drug screen, excitation contraction coupling, animal models, drug discovery, *C. elegans*, Mouse, Zebrafish

## Abstract

An multi-species approach can be used to identify small molecules with properties that might prove useful for the treatment of some neuromuscular diseases.

**Related research article** Volpatti JR, Endo Y, Knox J, Groom L, Brennan S, Noche R, Zuercher WJ, Roy P, Dirksen RT, Dowling JJ. 2020. Identification of drug modifiers for RYR1 related myopathy using a multi-species discovery pipeline. *eLife* **9**:e52946. doi: 10.7554/eLife.52946

Muscle contraction is a complicated process that starts with an electrical signal called an action potential entering a muscle cell, and ends with thin filaments sliding past thick filaments to make the cell shorter. A central part of this process involves the action potential activating a protein called a dihydropyridine (DHP) receptor, which then activates a calcium-ion channel called a ryanodine receptor. The activation of this channel in an organelle called the sarcoplasmic reticulum results in the release of calcium ions (Ca^2+^) into the cytoplasm of the cell. These ions go on to activate muscle contraction through a mechanism that involves promoting the interaction between thick and thin filaments (see Figure 1A).

**Figure 1.**
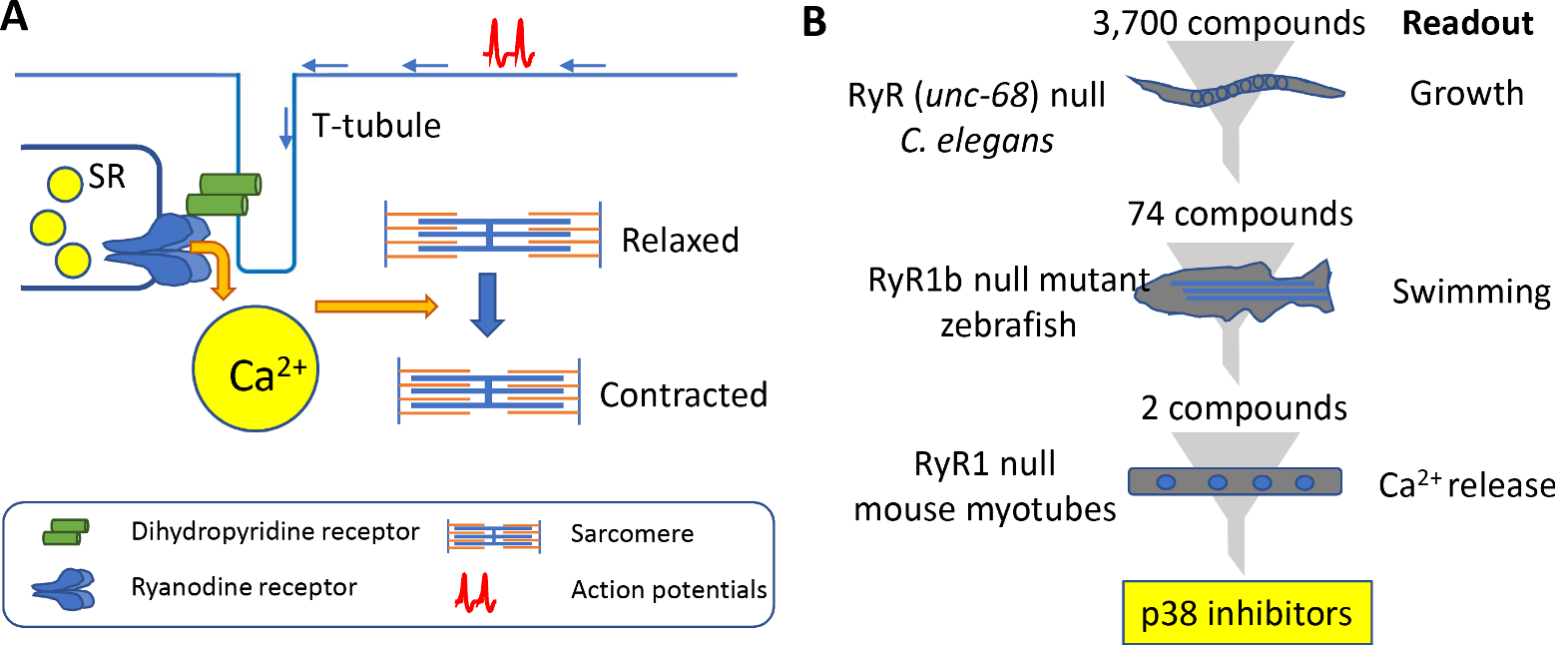
Muscle contraction and a 'multi-species discovery pipeline' for drug screening. (**A**) Ryanodine receptor 1 (RyR1; blue) is a channel protein that releases calcium ions (yellow) from the sarcoplasmic reticulum (SR) when activated by a dihydropyridine receptor (DHP; green). Muscle tissue contains thick filaments (blue) and thin filaments (orange) arranged in units called sarcomeres. The calcium ions cause structures on the thick filaments called myosin heads (not shown) to bind to the thin filaments and pull them so that the muscle contracts. The DHP receptor is activated by action potentials (red spikes) travelling along a structure called a T-tubule (blue). (**B**) Drug screening was performed sequentially using null mutants in *C. elegans* (top), zebrafish (middle), and mouse myotubes (bottom). A screen of 3700 compounds in *C. elegans* identified 74 compounds that enabled the mutants to grow. Testing many of these 74 compounds on zebrafish revealed two compounds that improved the swimming performance of the mutant animals. Both compounds were known to be inhibitors of a protein kinase called p38, and both were found to induce the release of calcium ions in mutant mouse muscle cells.

Congenital muscle diseases in humans can result from mutations in at least 20 genes, but mutations in the gene for ryanodine receptor 1 (RyR1) are the most common cause of such myopathies (Jungbluth et al., 2018; Robinson et al., 2006). Mutations in this gene cause malignant hyperthermia (a rare severe reaction that can occur during anesthesia), central core disease and a range of other myopathies that can result in severe disabilities and early mortality. There are currently no effective therapies for any of these conditions. Now, in eLife, James Dowling (Hospital for Sick Children and the University of Toronto) and colleagues – including Jonathan Volpatti as first author – report how they have used a 'multi-species discovery pipeline' to identify two compounds that might be effective in treating these patients (Volpatti et al., 2020).

The pipeline involved three species: the worm *C. elegans*, the zebrafish and mouse cells (see Figure 1B). *C. elegans* has just one type of ryanodine receptor (humans have three) and mutants that lack *unc-68*, the gene for this receptor, move much less than wild-type worms (Maryon et al., 1996). To make the phenotype more robust, Volpatti et al. exposed the mutant animals to a DHP inhibitor called nemadipine-A that induces larval growth arrest in mutant animals but not in wild-type animals. The researchers screened 3700 compounds to identify those that permitted mutant worms that had been exposed to nemadipine-A to reach adulthood. Initially, the screen revealed 278 compounds, but this number dropped to 74 after additional testing. Compounds that inhibited a protein kinase called p38 were over-represented in this sample. To explore if off-target effects might be responsible for their results, Volpatti et al. used RNAi to knock-down orthologs of p38 in worms. Again the mutant worms reached adulthood, confirming that the results were likely due to p38 being inhibited and not due to off-target effects.

Many of the 74 compounds identified from the worm screen were then tested for their ability to improve the movement of zebrafish that lacked a gene called *ryr1b* that encodes one of the ryanodine receptors: these mutants are normally poor swimmers (Hirata et al., 2007). Two of the p38 inhibitors were successful.

Volpatti et al. then moved to a mouse cell line called C2C12. These cells are myoblasts that can be differentiated into mature functional muscle cells, and the researchers used CRISPR/Cas9 to create mutants in which the gene *Ryr1* had been knocked out. When caffeine is administered to wild-type C2C12 cells they release calcium ions, but this does not happen with the mutant cells. However, when the mutant cells were treated with either of the p38 inhibitors, they released calcium ions. The researchers verified that the two compounds were likely specifically inhibiting p38 by observing the release of calcium ions from the knockout cells when any one of the three isoforms of p38 were knocked down by siRNA.

The role of p38 during the formation of muscle tissue has been studied intensively. p38 activates various factors that regulate muscle formation (Keren et al., 2006); it also activates adult muscle stem cells and promotes their self-renewal (Jones et al., 2005). Thus, p38 has been proposed as a potential therapeutic target for muscular dystrophies. Activation of p38 has been detected in mouse models of two forms of muscular dystrophy (Duchenne Muscular Dystrophy and Limb Girdle Muscular Dystrophy R6). Moreover, deletion of the gene for the alpha isoform of p38 results in reduced myopathy in both muscular dystrophy models via the inhibition of apoptosis (Wissing et al., 2014). Although the role of p38 in the pathology of the various myopathies related to RyR1 is not explored by Volpatti et al., the inhibition of apoptosis or the regulation of muscle formation may not be relevant, since their results were obtained on fully differentiated muscle cells. A more likely explanation is that p38 inhibition results in increased expression or activity of other Ca^2+^ channels.

A weakness of this study is that the screens were conducted on models that completely lack expression of RyR1, whereas none of the patients with malignant hyperthermia or RyR1-related myopathies are nulls. Recognizing this problem, Volpatti et al. plan to re-do their screens on mice or cells carrying missense mutations analogous to those found in malignant hyperthermia (Lopez et al., 2018), or the more complex mutations found in patients with RyR1-related myopathies (see, for example, Brennan et al., 2019). An intriguing question is how can the inhibition of p38 result in the release of more Ca^2+^ in animals or cells that have no RyR1 expression? As mentioned in the previous paragraph, it is possible that the inhibition of p38 increases the release of Ca^2+^ from the sarcoplasmic reticulum through other Ca^2+^channels such as the IP3R and STIM1/ORAI1 channels (which are found in both *C. elegans* and C2C12 cells), and RyR3 channel, which is also found in C2C12 cells.

Volpatti et al. conducted their drug screens rapidly and in mutant animals displaying phenotypes relevant to those found in human patients. For example, the screen of 3700 compounds in *C. elegans* was completed in just two weeks.

It is likely that their strategy of screening in *C. elegans* first, followed by zebrafish and cultured cells, could be used to screen drugs for many human genetic diseases that are due to mutations in proteins with orthologs in *C. elegans*. (About 40% of *C. elegans* proteins have human orthologs). This is particularly true for human diseases that display developmental or neuromuscular defects, as development is fast (fertilized egg to adult in three days) and occurs in distinct stages (embryo, four larval stages and adult), and there are powerful methods for studying the locomotion of these animals (see, for example, Restif et al., 2014). This presages another case in which worms will lead the way!
